# Investigating the relationship between Epstein‐Barr virus infection and gastric cancer: A systematic review and meta‐analysis

**DOI:** 10.1002/hsr2.1976

**Published:** 2024-03-18

**Authors:** Saman Dokanei, Dariush Minai‐Tehrani, Mohsen Moghoofei, Mosayeb Rostamian

**Affiliations:** ^1^ Faculty of Life Sciences and Biotechnology Shahid Beheshti University (GC) Tehran Iran; ^2^ Department of Microbiology, Faculty of Medicine Kermanshah University of Medical Sciences Kermanshah Iran; ^3^ Infectious Diseases Research Center, Health Institute Kermanshah University of Medical Sciences Kermanshah Iran; ^4^ Student Research Committee Kermanshah University of Medical Sciences Kermanshah Iran

**Keywords:** Epstein‐Barr virus, gastric cancer, meta‐analysis, systematic review

## Abstract

**Background and Aims:**

Gastric cancer (GC) is a common cancer type worldwide, and various factors can be involved in its occurrence. One of these factors is Epstein‐Barr virus (EBV) infection. In this regard, a systematic review and meta‐analysis was conducted to achieve a better understanding of the EBV prevalence in GC samples.

**Methods:**

English databases were searched and studies that reported the prevalence and etiological factors of EBV related to GC from July 2007 to November 2022 were retrieved. The reported data were selected based on the inclusion and exclusion criteria. The pooled prevalence of EBV infection with 95% confidence intervals was calculated. Quality assessment, heterogeneity testing, and publication bias assessment were also performed. The literature search showed 953 studies, of which 87 studies met our inclusion criteria and were used for meta‐analysis.

**Results:**

The pooled prevalence of EBV infection related to GC was estimated to be 9.5% (95% confidence interval [CI]: 8.2%−11%) in the general population. The prevalence of EBV infection related to GC by gender was 13.5% (95% CI: 11.1%−16.3%) in males and 7.6% (95% CI: 5.4%−10.6%) in females. No significant differences were observed in terms of geographical region. Out of the 87 studies included in the meta‐analysis, the most common diagnostic test was in situ hybridization (58 cases).

**Conclusions:**

Altogether, the results indicated that EBV infection is one of the important factors in the development of GC. However, this does not necessarily mean that EBV infection directly causes GC since other factors may also be involved in the development of GC. Therefore, it is recommended to conduct extensive epidemiological studies on various aspects of the relationship between this virus and GC, which can provide valuable information for understanding the relationship between EBV and GC.

## 1 INTRODUCTION AND OBJECTIVES

Gastric cancer (GC) poses a significant challenge to global health due to its prevalence as the fifth most frequently occurring cancer globally and its role as the third leading cause of cancer‐related deaths. GC is accountable for nearly 800,000 fatalities annually.^1^ The prevalence of this ailment is significantly higher in males than in females, occurring almost twice as frequently in the former. Additionally, the majority of cases of this disease materialize after the age of 60.^1^, ^2^ The occurrence of GC exhibits significant variation across different geographic regions, with the highest prevalence observed in East Asia, certain regions of Eastern Europe, and South American nations. The lowest prevalence is noted in North America and Africa. Furthermore, developing countries bear the brunt of this disease, accounting for over 70% of its global burden.^3^

Before the 1980s, GC held the position of the primary cause of cancer‐related deaths globally.^4^ Over the last 50 years, a marked reduction in the prevalence of GC has been observed. This decline is primarily attributed to advancements in food storage and preservation, decreased consumption of salty and smoked foods, and possibly a reduction in the prevalence of Helicobacter pylori infection.^5^, ^6^ Adenocarcinomas comprise more than 95% of all GC cases and are typically categorized based on their anatomical location (proximal/cardia or distal/noncardia) and histological type (intestinal or diffuse).^7^ Apart from infections, certain dietary habits have been identified as risk factors for GC, including high consumption of red meat, smoked food, and salt, as well as low intake of fruits and vegetables.^8^

Various types of gastric tumors can be identified based on their molecular characteristics, such as EBV‐positive tumors, tumors exhibiting microsatellite instability, chromosomally stable tumors, and tumors with chromosomal instability.^9^

Epstein‐Barr virus (EBV), commonly referred to as the human herpesvirus 4, was discovered by Barr and Epstein in 1964 through electron microscopy of African Burkitt lymphoma cell suspensions.^10^ This virus was the first human oncogenic virus to be discovered.^11^ The initial documentation of EBV's involvement in GC occurred in 1990 when it was reported in gastric lymphoepithelial carcinoma. Since then, numerous investigations have been carried out on EBV and its correlation with various cancers, including GC.^12^

Serological responses provide evidence of prior infection with EBV, which is present in over 90% of adults worldwide. Exposure to this virus typically occurs early in life, and most children in developing countries show positive serum presence of the virus by the age of five. While the onset of infection is delayed in areas with greater social and economic development, adults are almost equally positive for EBV. EBV is mainly transmitted through contact with respiratory secretions, which increases access and entry into the reticuloendothelial cells of the upper respiratory tree. EBV primarily targets B lymphocytes, however in immunocompromised hosts, it can infect other cell types, including epithelial cells. EBV is associated with several diseases, including T‐cell lymphoma, NK‐cell leukemia, and lymphoproliferative disorders.^13^, ^14^, ^15^ While EBV has mechanisms to evade the immune system, a robust immune system can keep the virus in check through cellular immunity, including T cells, NK cells, and NKT cells. The precise molecular mechanisms behind how EBV induces latency rather than cell death are not entirely clear, but it is known that the virus employs latency mechanisms to evade the immune system.^16^

There have been various investigations carried out on the correlation between GC and EBV, but more studies are needed to clarify this correlation. As a result, one of the most effective approaches to obtaining the most accurate results is to conduct meta‐analytic studies, which amalgamate the available evidence and offer a comprehensive estimation of reality from diverse angles. Several meta‐analytic studies have been undertaken to examine the association between EBV infection and GC.^17^, ^18^, ^19^ However, it seems that there is a need for more comprehensive studies in this field that can offer a more accurate estimation of reality by combining the results of these studies. This would enable better decision‐making for therapeutic purposes and health policies in the domain of health care and treatment.

To fill this research gap, a systematic review was conducted to investigate published studies that explore the prevalence of EBV in samples of GC from different parts of the world. The main objective of this review was to carefully analyze the existing evidence systematically to gain a more comprehensive understanding of the association between EBV and GC. The ultimate aim of this endeavor is to equip health care professionals and policymakers with valuable insights that can inform clinical decision‐making and health policies regarding the prevention, diagnosis, and treatment of GC.

## 2 METHODS

### 2.1 Data sources

The study was conducted following the PRISMA* guidelines.^20^ A systematic search strategy was applied to identify relevant texts on the prevalence of EBV associated with GC in electronic databases including PubMed, Science Direct, Scopus, Google Scholar, and Web of Science, from July 2007 to November 2022 using appropriate keywords such as “Gastric cancer OR Epstein‐Barr virus” OR “Gastric cancer AND EBV” OR “Gastric cancer AND Epstein‐Barr virus” OR “stomach cancer AND EBV” OR “stomach neoplasms” OR “Neoplasm, Gastric” OR “Epstein‐Barr Virus” OR “Gastric.”

### 2.2 Study eligibility criteria

Inclusion criteria were as follows: studies related to GC and EBV, original articles, published between 2007 and 2022, studies that mentioned the EBV diagnostic test, and articles published in English.

Exclusion criteria were as follows: reviewed studies, case reports, letters, studies that evaluated the presence of EBV in patients with underlying disorders, studies with insufficient data, studies in languages other than English, studies published before 2007, and studies that did not declare the EBV diagnostic test.

### 2.3 Data collection

After the initial screening based on the inclusion and exclusion criteria, two researchers independently extracted data from all eligible studies using a pre‐designed form in Microsoft Excel 2013. A list of abstracts was prepared by the researchers. Studies that were irrelevant to the research question were excluded, and relevant or potentially relevant studies were included in the initial list. Any discrepancies between the two researchers were resolved through consultation, and if necessary, a third researcher was consulted to make the final decision. By reviewing the full text of the remaining articles, studies that were completely relevant to the research objective were selected.

### 2.4 Quality assessment

The quality of studies was checked using the Appraisal Critical checklist from the Joanna Briggs Institute (JBI),^21^ which contains nine questions related to the quality assessment of cross‐sectional and prevalence studies. This checklist includes questions about the sample size, study groups, study setting, and statistical analysis. Each question is assigned a score, and studies that score 6 or higher are included in the analysis. Quality assessment was independently performed by two authors, and any discrepancies were resolved through consultation with a third author.

### 2.5 Statistical analysis

Statistical analysis was performed using Comprehensive Meta‐analysis (CMA)† software. The standard error for the desired outcomes in the studies with a 95% confidence level for prevalence was calculated. The Cochrane Q test and the I‐squared index were used to assess heterogeneity among the studies. Heterogeneity less than 20% is considered low, between 20% and 60% is considered moderate, and greater than 60% is considered high. Depending on the heterogeneity of the studies and the significance of the heterogeneity index (I‐squared), a fixed or random‐effects model was used in the meta‐analysis. Subgroup analysis was used to compare the prevalence of EBV‐associated GC among different groups, such as gender, geographic regions, diagnostic methods, and sample types. A meta‐regression analysis was done to investigate the relationship between the prevalence of EBV and the sampling year. Publication bias was assessed using the Egger test (significance at *p* < 0.05).

## 3 RESULTS

### 3.1 Characteristics of the study

**Figure 1:**
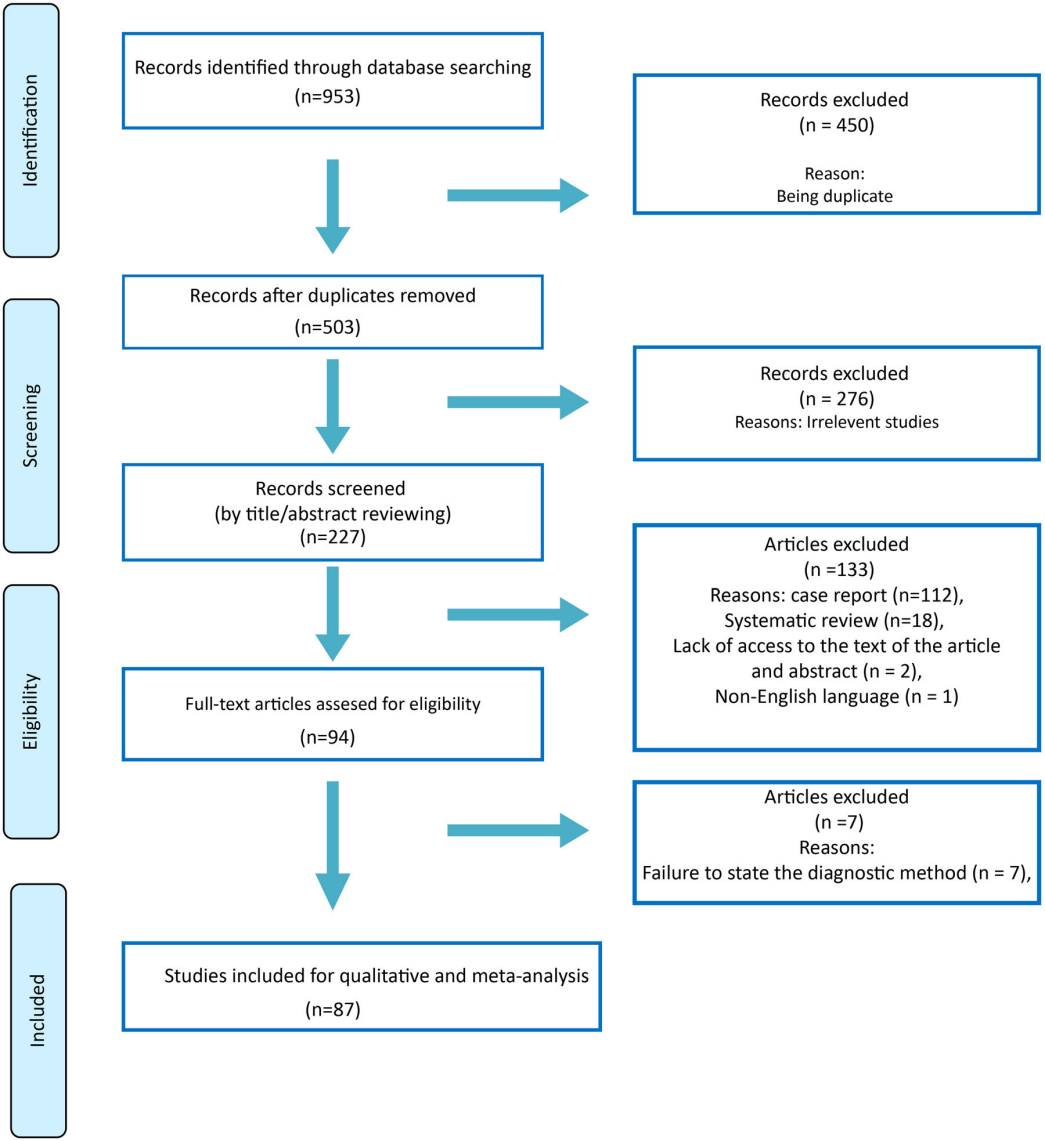
Flowchart of study selection.

After a systematic search in electronic databases and using reference lists of articles, 87 human studies on the impact of EBV infection on GC were eligible (Figure 1). The characteristics of 87 studies involving 30,242 participants have been included (Table 1). The studies conducted in 22 countries from almost all continents. Diagnostic methods used were 58 cases of in situ hybridization, three cases of immunohistochemistry‐in situ hybridization, two cases of in situ fluorescent hybridization, seven cases of chromogenic in situ hybridization, eight cases of in situ polymerase chain reaction hybridization, and nine cases of various polymerase chain reaction methods.

**Table 1:** Baseline characteristics of included studies for EBV‐associated gastric cancer.

Authors	Country	Year of publication	Sampling year (median)	Sample size	Number of EBV	Male+^a^	Female+^a^	Method
Abdirad et al.^22^	Iran	2007	1986.5	273	9	8	1	ISH
Aslane et al.^23^	Algeria	2016	2011.5	97	22	18	4	FISH
Akkus et al.^24^	Turkey	2022	N/A	25	8	N/A	N/A	QPCR
Aversa et al.^25^	China	2021	2001	1035	22	19	3	ISH
Baek et al.^26^	South Korea	2018	2014	276	59	50	9	ISH
Birkman et al.^27^	Finland	2018	2002.5	238	17	15	2	lHC‐lSH
Böger et al.^28^	Germany	2017	N/A	484	22	20	2	ISH
Boysen et al.^29^	Denmark	2011	1991	131	10	N/A	N/A	lHC‐lSH
Boysen et al.^30^	Denmark	2009	1987.5	212	18	15	3	lHC‐lSH
Camargo et al.^31^	Latvia	2018	2012.5	302	28	N/A	N/A	ISH
Camargo et al.^32^	USA	2014	1997.5	2648	184	N/A	N/A	ISH
Castaneda et al.^33^	Peru	2019	2016.5	375	72	N/A	N/A	QPCR
Chen et al.^34^	China	2010	2003	676	45	37	8	ISH
Cheng et al. −1^35^	China	2015	2003	53	8	7	1	ISH
Cheng et al. −2^36^	China	2021	2007	846	42	38	4	ISH
Choi et al.^37^	South Korea	2020	2008.5	514	32	24	8	ISH
Ciarpaglini et al.^38^	Spain	2019	2010	209	13	10	3	ISH
Dai et al.^39^	China	2016	N/A	398	10	N/A	N/A	ISH
de Lima et al.^40^	Brazil	2012	N/A	160	11	9	1	PCR‐ISH
de Rosa et al.^41^	ltaly	2018	1996	169	33	24	9	ISH
de Souza et al.^42^	Brazil	2018	2010	302	62	43	19	ISH
Faghihloo et al.^43^	lran	2014	2009	90	6	4	2	qRT‐PCR
Fang et al.^44^	Taiwan	2020	2008.5	460	43	35	8	ISH
Ferrasi et al.^45^	Brazil	2010	N/A	54	5	N/A	N/A	ISH
Gasenko et al.^46^	Latvia	2019	2012.5	302	26	22	4	ISH
Guerfali et al.^47^	Tunisia	2011	2004	81	12	6	6	ISH
Gullo et al.^48^	Portugal	2019	N/A	78	19	N/A	N/A	CISH
Guo et al.^49^	China	2020	N/A	270	18	14	4	PCR‐ISH
Huang et al.^50^	Taiwan	2019	2003	1248	65	54	11	ISH
Irkkan et al.^51^	Turkey	2017	2009.5	105	8	8	N/A	ISH
Jung et al.^52^	South Korea	2007	1992	111	7	6	1	ISH
Kang et al.^53^	South Korea	2016	N/A	1318	120	96	24	ISH
Kawazoe et al. −1^54^	Japan	2017	2006	487	25	N/A	N/A	ISH
Kawazoe et al. −2^55^	Japan	2019	2016	225	14	11	N/A	CISH
Kim et al.−1^56^	South Korea	2010	2004	247	18	17	1	ISH
Kim et al.−2^57^	South Korea	2017	N/A	207	13	N/A	N/A	ISH
Kim et al.−3^58^	South Korea	2019	2005.5	273	25	N/A	N/A	ISH
Kim et al.−4^59^	USA	2019	2011	43	6	5	1	ISH
Kim et al.−5^60^	South Korea	2019	2012.5	297	22	N/A	N/A	CISH
Kim et al.−6^61^	South Korea	2020	2011	286	17	N/A	N/A	ISH
Kim et al.−7^62^	USA	2021	2007.5	88	5	N/A	N/A	lHC‐lSH
Koriyama et al. −2^63^	Japan	2010	1992.5	156	21	N/A	N/A	ISH
Koriyama et al. −1^64^	Japan	2007	1984	179	59	N/A	N/A	ISH
Ksiaa et al.^65^	Tunisia	2014	N/A	43	4	4	N/A	PCR‐ISH
Leila et al.^66^	lran	2016	2011.5	90	10	9	1	RT‐PCR
Lima et al.^67^	Brazil	2008	N/A	71	6	6	N/A	ISH
Liu et al.^68^	China	2016	2011	206	15	10	5	ISH
Ma et al.−1^7^	USA	2016	N/A	44	7	6	1	ISH
Ma et al.−2^69^	China	2017	2008.5	571	31	26	5	ISH
Martinson et al.^70^	USA	2020	2010	85	19	13	6	CISH
Moore et al.^71^	Germany	2020	2006.5	74	17	N/A	N/A	PCR
Na et al.^72^	South Korea	2017	2015	205	15	13	2	ISH
Nogueira et al.^73^	Portugal	2017	N/A	82	9	8	1	PCR‐ISH
Noh et al.^74^	South Korea	2018	2005.5	479	36	N/A	N/A	ISH
Nshizirungu et al.^75^	Morocco	2021	2016	97	6	6	0	ISH
Nunes et al.^76^	Brazil	2021	2010	1000	190	127	63	ISH
Osumi et al.^77^	Japan	2019	2009.5	898	71	58	13	ISH
Pereira et al.^78^	Brazil	2018	2012.5	286	30	23	7	ISH
Pinto et al.^79^	ltaly	2020	2015.5	70	2	1	1	CISH
Ribeiro et al.^80^	Portugal	2019	2009	199	5	3	2	ISH
Ribeiro et al.^81^	Portugal	2017	2011	179	15	12	3	ISH
Roh et al.^82^	South Korea	2019	N/A	582	41	38	3	ISH
Rihane et al.^83^	Morocco	2021	N/A	100	40	22	18	nested PCR
Ryan et al.^84^	USA	2009	N/A	113	11	N/A	N/A	QPCR‐lSH
Saito et al.^85^	Japan	2017	2001.5	232	96	76	20	ISH
Setia et al.^86^	USA/South Korea	2019	1998	486	33	N/A	N/A	ISH
Shen et al.^87^	China	2017	2009	202	42	29	13	PCR
Siciliano et al.^88^	ltaly	2022	2010	40	7	5	2	ISH
Song et al.^89^	South Korea	2010	2001	1080	123	91	32	ISH
Sun et al.^90^	China	2019	2013	165	2	1	1	ISH
Sundar et al.^91^	South Korea	2018	2006	220	71	62	9	ISH
Trimeche et al.^92^	Tunisia	2009	2001.5	96	4	4	N/A	PCR‐ISH
Truong et al.^93^	USA	2009	1996.5	235	12	11	1	ISH
Tsai et al.^94^	Taiwan	2017	2003	1039	52	42	10	ISH
Ushiku et al. −1^95^	Japan	2007	1997	51	13	N/A	N/A	ISH
Ushiku et al. −2^96^	Japan	2015	N/A	54	10	9	1	ISH
Valentini et al.^97^	ltaly	2019	2015.5	70	2	N/A	N/A	CISH
Wanvimonsuk et al.^98^	Thailand	2019	N/A	33	4	N/A	N/A	PCR‐ISH
Yanagi et al.^99^	Japan	2019	2012	1067	69	61	8	ISH
Yang et al.^100^	China	2021	2013.5	226	13	N/A	N/A	ISH
Yoon et al.^101^	Canada	2019	2006	107	3	2	1	ISH
Zhang et al.−1^102^	China	2016	2010	600	30	25	5	ISH
Zhang et al.−2^103^	China	2019	2004	1013	58	N/A	N/A	FISH
Zhao et al.^104^	Hong kong	2012	2002.5	555	68	60	8	PCR‐ISH
Zhou et al.^105^	China	2019	2016	300	28	N/A	N/A	ISH
Zhu et al.^106^	China	2013	2008.5	58	13	N/A	N/A	RT‐PCR
Zebardast et al.^107^	lran	2022	2015	81	13	8	5	qRT‐PCR

^a^ Gastric cancer patients positive for EBV.

Abbreviations: CISH, Chromogenic in situ hybridization; FISH, Fluorescence In Situ Hybridization; IHC, immunohistochemistry; ISH, In Situ Hybridization; N/A, not available; PCR, Polymerase Chain Reaction; median, Based on the beginning and end of the year the sampling of the studies is set.

### 3.2 Prevalence of EBV associated with GC

The prevalence of EBV in patients with GC in each study as well as the pooled prevalence of all studies are shown in the general population (A), female (B), and male subjects (C).

Using the number of EBV in GC samples, the prevalence of EBV was calculated for each study as well as the total studies in the general population. The overall pooled prevalence of EBV in GC samples in the general population was 9.5% (95% confidence interval [CI]: 8.2%−11.0%: *I* ^2^ = 92.9%) (*p* = 0.000), (Table 2).

**Table 2:** Prevalence of EBV in gastric cancer samples.

Study name	Prevalence in the general population				Prevalence in female				Prevalence in male			
	Event rate	Lower limit	Upper limit	*p* Value	Event rate	Lower limit	Upper limit	*p* Value	Event rate	Lower limit	Upper limit	*p* Value
Abdirad et al.	0.033	0.017	0.062	0.000	0.013	0.002	0.088	0.000	0.043	0.021	0.083	0.000
Aslane et al.	0.227	0.154	0.321	0.079	0.133	0.051	0.306	0.000	0.269	0.176	0.387	0.000
Akkus S	0.320	0.169	0.522	0.000	N/A	N/A	N/A	N/A	N/A	N/A	N/A	N/A
Aversa JG	0.021	0.014	0.032	0.000	N/A	N/A	N/A	N/A	0.023	0.015	0.037	0.000
Baek et al.	0.214	0.169	0.266	0.000	0.120	0.064	0.215	0.000	0.249	0.194	0.313	0.000
Birkman et al.	0.071	0.045	0.112	0.000	0.014	0.004	0.054	0.000	0.149	0.092	0.232	0.000
Boger et al.	0.045	0.030	0.068	0.000	0.011	0.003	0.043	0.000	0.066	0.043	0.100	0.000
Boysen et al.	0.076	0.042	0.136	0.000	0.039	0.013	0.114	0.000	0.111	0.068	0.176	0.000
Boysen T	0.085	0.054	0.131	0.000	N/A	N/A	N/A	N/A	N/A	N/A	N/A	N/A
Camargo MC	0.093	0.065	0.131	0.000	N/A	N/A	N/A	N/A	N/A	N/A	N/A	N/A
Camargo et al.	0.069	0.060	0.080	0.000	N/A	N/A	N/A	N/A	N/A	N/A	N/A	N/A
Castaneda et al.	0.192	0.155	0.235	0.000	N/A	N/A	N/A	N/A	N/A	N/A	N/A	N/A
Chen et al.	0.067	0.050	0.088	0.000	0.033	0.017	0.065	0.000	0.085	0.062	0.115	0.000
Cheng et al. −1	0.151	0.077	0.274	0.000	0.125	0.017	0.537	0.000	0.156	0.076	0.292	0.000
Cheng et al. −2	0.050	0.037	0.067	0.000	0.015	0.006	0.040	0.000	0.065	0.048	0.088	0.000
Choi et al.	0.062	0.044	0.087	0.000	0.048	0.024	0.093	0.000	0.069	0.047	0.101	0.000
Ciarpaglini et al.	0.062	0.036	0.104	0.000	0.053	0.017	0.151	0.000	0.069	0.038	0.124	0.000
Dai et al.	0.025	0.014	0.046	0.000	N/A	N/A	N/A	N/A	N/A	N/A	N/A	N/A
de Lima et al.	0.069	0.038	0.120	0.000	N/A	N/A	N/A	N/A	0.950	0.525	0.997	0.042
de Rosa et al.	0.195	0.142	0.262	0.000	0.143	0.076	0.252	0.000	0.233	0.161	0.324	0.000
de Souza et al.	0.205	0.163	0.255	0.000	0.178	0.116	0.262	0.000	0.221	0.168	0.284	0.000
Faghihloo E,	0.067	0.030	0.141	0.000	0.077	0.019	0.261	0.001	0.063	0.024	0.155	0.000
Fang et al.	0.093	0.070	0.124	0.000	0.061	0.031	0.117	0.000	0.106	0.077	0.145	0.000
Ferrasi et al.	0.093	0.039	0.204	0.000	N/A	N/A	N/A	N/A	N/A	N/A	N/A	N/A
Gasenko et al.	0.086	0.059	0.123	0.000	0.022	0.008	0.057	0.000	0.185	0.125	0.265	0.000
Guerfali et al.	0.148	0.086	0.243	0.000	0.182	0.084	0.350	0.000	0.125	0.057	0.252	0.000
Gullo et al.	0.244	0.161	0.351	0.000	N/A	N/A	N/A	N/A	N/A	N/A	N/A	N/A
Guo et al.	0.067	0.042	0.103	0.000	0.077	0.029	0.188	0.000	0.064	0.038	0.000	0.000
Huang et al.	0.052	0.041	0.066	0.000	0.023	0.013	0.042	0.000	0.069	0.054	0.090	0.000
Irkkan et al.	0.076	0.039	0.145	0.000	N/A	N/A	N/A	N/A	0.104	0.053	0.194	0.000
Jung et al.	0.063	0.030	0.126	0.000	0.029	0.004	0.177	0.000	0.079	0.036	0.165	0.000
Kang et al.	0.091	0.077	0.108	0.000	0.980	0.749	0.999	0.000	0.995	0.923	1.000	0.000
Kawazoe et al. −1	0.051	0.035	0.075	0.000	N/A	N/A	N/A	N/A	N/A	N/A	N/A	N/A
Kawazoe et al. −2	0.062	0.037	0.102	0.000	N/A	N/A	N/A	N/A	0.081	0.045	0.140	0.000
Kim et al.−1	0.073	0.046	0.113	0.000	N/A	N/A	N/A	N/A	0.972	0.678	0.998	0.013
Kim et al.−2	0.063	0.037	0.105	0.000	N/A	N/A	N/A	N/A	N/A	N/A	N/A	N/A
Kim et al.−3	0.092	0.063	0.132	0.000	N/A	N/A	N/A	N/A	N/A	N/A	N/A	N/A
Kim et al.−4	0.140	0.064	0.278	0.000	0.053	0.007	0.294	0.005	0.208	0.089	0.413	0.008
Kim et al.−5	0.074	0.049	0.110	0.000	N/A	N/A	N/A	N/A	N/A	N/A	N/A	N/A
Kim et al.−6	0.059	0.037	0.094	0.000	N/A	N/A	N/A	N/A	N/A	N/A	N/A	N/A
Kim et al.−7	0.057	0.024	0.129	0.000	N/A	N/A	N/A	N/A	N/A	N/A	N/A	N/A
Koriyama et al. −2	0.135	0.089	0.198	0.000	N/A	N/A	N/A	N/A	N/A	N/A	N/A	N/A
Koriyama et al. −1	0.330	0.265	0.402	0.000	N/A	N/A	N/A	N/A	N/A	N/A	N/A	N/A
Ksiaa et al.	0.093	0.035	0.223	0.000	N/A	N/A	N/A	N/A	0.148	0.057	0.335	0.001
Leila Z	0.111	0.061	0.194	0.000	N/A	N/A	N/A	N/A	0.105	0.048	0.215	0.000
Lima et al.	0.085	0.038	0.176	0.000	0.059	0.008	0.320	0.001	0.123	0.065	0.220	0.000
Liu et al.	0.073	0.044	0.117	0.000	0.094	0.040	0.207	0.000	0.065	0.036	0.117	0.000
Ma et al.−1	0.159	0.078	0.298	0.000	0.053	0.007	0.294	0.005	0.240	0.112	0.442	0.014
Ma et al.−2	0.054	0.038	0.076	0.000	0.030	0.013	0.071	0.000	0.064	0.044	0.092	0.000
Martinson et al.	0.224	0.147	0.324	0.000	0.182	0.084	0.350	0.001	0.250	0.151	0.384	0.001
Moore et al.	0.230	0.148	0.339	0.000	N/A	N/A	N/A	N/A	N/A	N/A	N/A	N/A
Na et al.	0.073	0.045	0.118	0.000	0.034	0.008	0.126	0.000	0.089	0.052	0.147	0.000
Nogueira et al.	0.110	0.058	0.198	0.000	0.027	0.004	0.168	0.000	0.178	0.091	0.317	0.000
Noh et al.	0.075	0.055	0.102	0.000	N/A	N/A	N/A	N/A	N/A	N/A	N/A	N/A
Nshizirungu et al.	0.062	0.028	0.131	0.000	0.013	0.001	0.175	0.002	0.102	0.046	0.208	0.000
Nunes et al.	0.190	0.167	0.216	0.000	0.184	0.147	0.229	0.000	0.193	0.165	0.225	0.000
Osumi et al.	0.079	0.063	0.099	0.000	0.043	0.025	0.073	0.000	0.097	0.076	0.123	0.000
Pereira et al.	0.105	0.074	0.146	0.000	0.059	0.028	0.118	0.000	0.138	0.093	0.199	0.000
Pinto et al.	0.029	0.007	0.107	0.000	0.042	0.001	0.244	0.002	0.022	0.003	0.139	0.000
Ribeiro et al.−1	0.025	0.010	0.059	0.000	0.024	0.006	0.089	0.000	0.026	0.009	0.078	0.000
Ribeiro et al.	0.084	0.051	0.134	0.000	0.042	0.014	0.123	0.000	0.111	0.064	0.186	0.000
Roh et al.	0.070	0.052	0.094	0.000	0.409	0.275	0.558	0.230	0.393	0.275	0.525	0.112
Rihane FE,	0.400	0.309	0.499	0.047	0.017	0.006	0.051	0.000	0.093	0.068	0.125	0.000
Ryan et al.	0.097	0.055	0.167	0.000	N/A	N/A	N/A	N/A	N/A	N/A	N/A	N/A
Saito et al.	0.414	0.352	0.478	0.009	0.976	0.713	0.999	0.009	0.994	0.905	1.000	0.000
Setia et al.	0.068	0.049	0.094	0.000	N/A	N/A	N/A	N/A	N/A	N/A	N/A	N/A
Shen et al.	0.208	0.157	0.269	0.000	0.206	0.124	0.324	0.000	0.209	0.149	0.284	0.000
Siciliano et al.	0.175	0.086	0.324	0.000	0.143	0.036	0.427	0.019	0.192	0.082	0.387	0.004
Song et al.	0.114	0.096	0.134	0.000	0.985	0.799	0.999	0.003	0.995	0.919	1.000	0.000
Sun et al.	0.012	0.003	0.047	0.000	0.021	0.003	0.134	0.000	0.009	0.001	0.058	0.000
Sundar et al.	0.323	0.264	0.387	0.000	0.950	0.525	0.997	0.042	0.992	0.885	1.000	0.001
Trimeche et al.	0.042	0.016	0.106	0.000	N/A	N/A	N/A	N/A	0.066	0.025	0.162	0.000
Truong et al.	0.051	0.029	0.088	0.000	N/A	N/A	N/A	N/A	0.958	0.575	0.997	0.030
Tsai et al.	0.050	0.038	0.065	0.000	0.024	0.013	0.044	0.000	0.068	0.051	0.091	0.000
Ushiku et al. −1	0.255	0.154	0.391	0.001	N/A	N/A	N/A	N/A	N/A	N/A	N/A	N/A
Ushiku et al. −2	0.185	0.103	0.311	0.000	N/A	N/A	N/A	N/A	0.950	0.525	0.997	0.042
Valentini et al.	0.029	0.007	0.107	0.000	N/A	N/A	N/A	N/A	N/A	N/A	N/A	N/A
Wanvimonsuk et al.	0.121	0.046	0.282	0.000	N/A	N/A	N/A	N/A	N/A	N/A	N/A	N/A
Yanagi et al.	0.065	0.051	0.081	0.000	0.024	0.012	0.047	0.000	0.083	0.065	0.105	0.000
Yang et al.	0.058	0.034	0.097	0.000	N/A	N/A	N/A	N/A	N/A	N/A	N/A	N/A
Yoon et al.	0.028	0.009	0.083	0.000	0.024	0.003	0.154	0.000	0.030	0.008	0.113	0.000
Zhang et al.−1	0.050	0.035	0.071	0.000	0.023	0.010	0.054	0.000	0.065	0.044	0.095	0.000
Zhang et al.−2	0.057	0.045	0.073	0.000	N/A	N/A	N/A	N/A	N/A	N/A	N/A	N/A
Zhao et al.	0.123	0.098	0.153	0.000	0.944	0.495	0.997	0.052	0.992	0.882	0.999	0.001
Zhou et al.	0.093	0.065	0.132	0.000	N/A	N/A	N/A	N/A	N/A	N/A	N/A	N/A
Zhu et al.	0.224	0.135	0.349	0.000	N/A	N/A	N/A	N/A	N/A	N/A	N/A	N/A
Zebardast et al.	0.160	0.096	0.257	0.000	0.208	0.089	0.413	0.000	0.140	0.072	0.256	0.000
Overall	0.095	0.082	0.110	0.000	0.076	0.054	0.106	0.000	0.135	0.111	0.163	0.000

Subgroup analysis of EBV infection associated with GC based on gender showed that the prevalence of EBV associated with GC in females was 7.6% (95% CI: 5.4%−10.6%, *I* ^2^ = 86.4%) (Table 2) while in males it was 13.5% (95% CI: 11.1%−16.3%, *I* ^2^ = 89.5%) (Table 2).

### 3.3 Prevalence of EBV associated with GC based on country, sample type, and methods

The subgroup analyses of EBV in the GC samples based on the country (A), sample type (B), and methods of detection (C) were illustrated.

**Figure 2:**
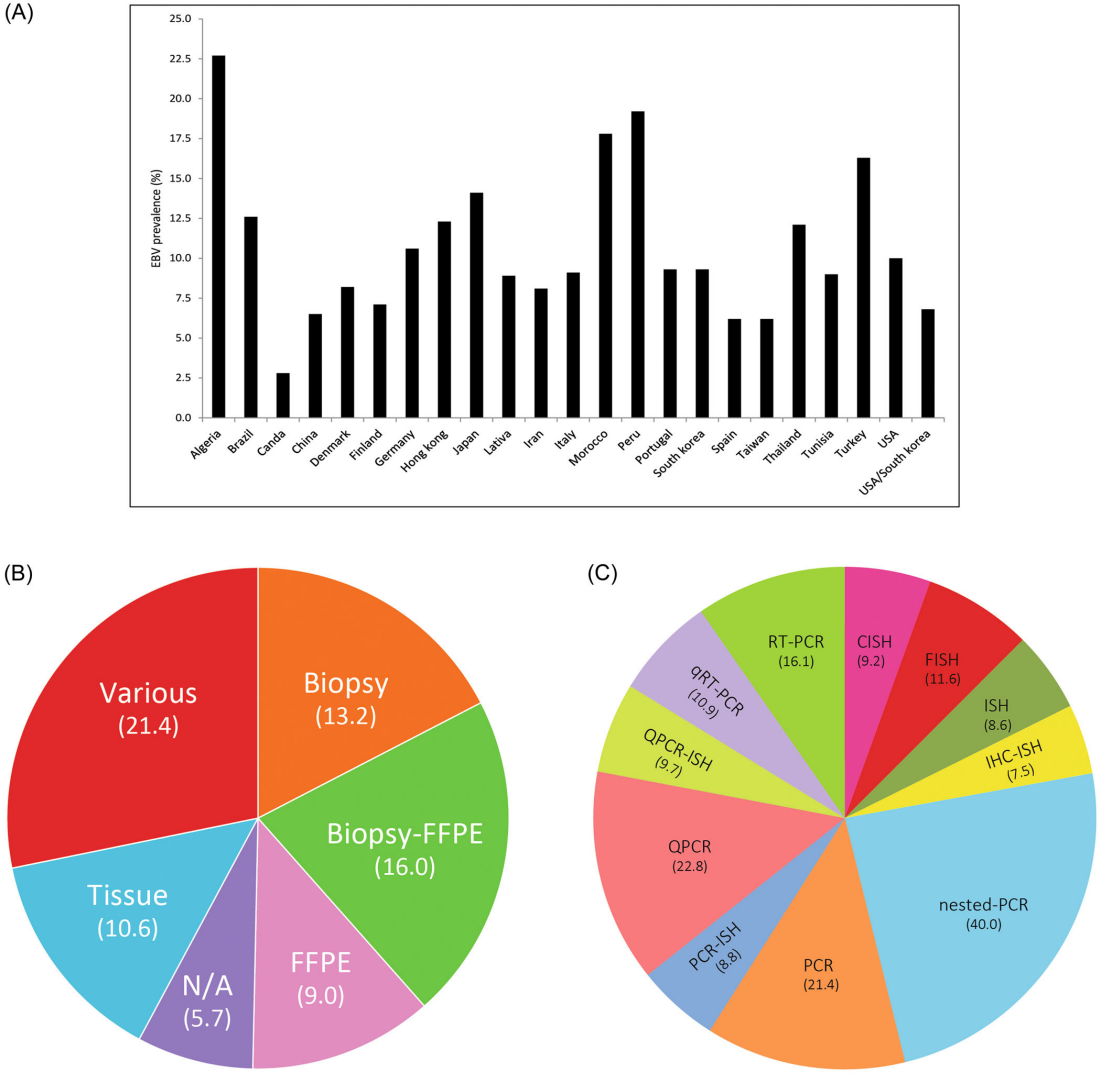
Charts of subgroup analyses on EBV in the gastric cancer samples. The subgroup analyses of EBV in the gastric cancer samples based on the country (A), sample type (B), and methods of detection (C) are illustrated. The numbers show the EBV prevalence (event rate) percentages. N/A means not available.

Subgroup analysis of EBV infection associated with GC based on geographical location (country), showed different prevalence ranges from 2.8% in Canada to 22.7% in Algeria (Figure 2A). It should be mentioned that the low prevalence in Canada entirely based on a single small study and has yet to be confirmed.

Subgroup analysis of EBV infection associated with GC based on the sample type showed a higher reported prevalence in biopsy (13.2%) than in formalin‐fixed and paraffin‐embedded (FFPE) (0.9%) (Figure 2B). Subgroup analysis based on diagnostic method, showed that in situ hybridization has been used the most. Meanwhile, the highest rate of prevalence is estimated by the PCR method (Figure 2C).

### 3.4 Prevalence of EBV associated with GC during years

**Figure 3:**
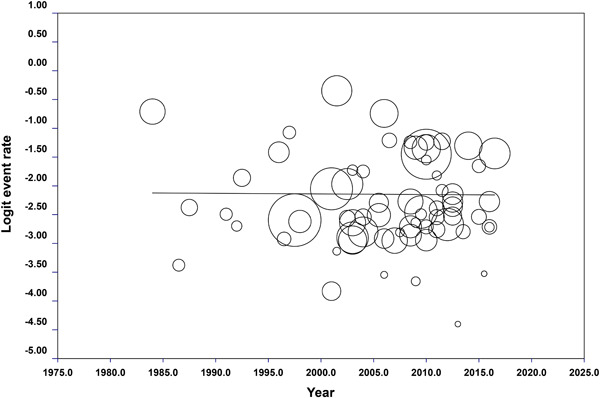
Meta‐regression plot of EBV in the gastric cancer samples during sampling years. As the plot shows there is no significant difference in overall EBV prevalence in GC samples over years of sampling. EBV, Epstein‐Barr virus; GC, gastric cancer.

A meta‐regression was conducted to assay the trend of EBV prevalence in GC samples during sampling years. Although we included studies from 2007 to 2022, the samplings have been performed since 1984 afterward. The results of meta‐regression showed no obvious increase or decrease in EBV prevalence in GC samples during the years (Figure 3).

### 3.5 Publication bias

The publication bias was assessed using the data of EBV prevalence in GC samples by performing Egger's test. The results showed the absence of publication bias in studies (Egger's test = 0.09942, *p* > 0.05).

## 4 DISCUSSION

Several meta‐analytic studies have investigated the connection between GC and EBV across the globe. However, there is a pressing need to update the information on the prevalence of EBV associated with GC and obtain an up‐to‐date estimate of how this virus behaves at a global level. To address this, the present study was conducted that aimed to provide a current estimate of the global prevalence of EBV linked to GC. The study found that the prevalence of EBV associated with GC is approximately 9.5%, indicating that a significant number of GC cases may be linked to EBV infection. In a study done by Hirabayashi et al. in 2023, the prevalence was estimated to be 7.5% in 68,000 patients.^108^ Tavakoli et al. reported a prevalence of 8.77% in 20,361 GC patients in 2020.^19^ Lee et al. reported a prevalence of 8.8% in 9,738 individuals in their meta‐analysis in 2009.^109^ Murphy et al. estimated a prevalence of 8.7% in 15,952 patients in 2009.^18^ Our study's findings are in agreement with these previous reports regarding the connection between EBV and GC. The prevalence rate of EBV associated with GC that we have estimated is significant and implies that EBV is a crucial risk factor for the development of a subset of GC.

However, it is important to note that while there is a strong correlation between EBV infection and GC, this does not necessarily imply that EBV directly causes GC. Other factors that can contribute to the development of this cancer and create conditions for the virus to impact its development should also be investigated and analyzed. Given the relatively high prevalence of EBV‐associated GC, it is crucial to conduct more extensive epidemiological studies. This would enable us to better understand the dynamics of this virus and the factors that influence its effects. By doing so, more comprehensive measures can be taken in terms of early warning, prevention, and control of this virus for communities. Therefore, it is important to develop effective strategies to mitigate the impact of EBV‐associated GC, improve public health outcomes, and prevent the spread of this disease.

According to our analyses, male patients diagnosed with GC have a higher likelihood, approximately 1.8 times, of being carriers of EBV compared to female patients diagnosed with GC. Li et al. estimated that EBV‐related cases of GC in males are 3.7 times more than in females.^110^ Qiao et al. reported that the prevalence of males with the disease is 4.2 times higher than that of females.^111^ Tavakoli et al. also reported that the prevalence of males with the disease is 1.9 times higher than that of females.^19^ The greater occurrence of EBV in male GC patients can be explained by various factors, including genetic, lifestyle, and occupational factors, as well as gender‐related factors such as sex hormones. However, it is challenging to ascertain whether the observed disparity in the prevalence of EBV in male GC patients compared to female GC patients is solely due to gender‐related factors or not.

The analysis of sample types revealed a higher frequency of EBV detection in biopsy samples compared to FFPE samples from GC patients, despite the larger number of FFPE samples available. The reason why most studies have relied on FFPE samples for diagnosis is likely due to the widespread use of FFPE tissues for clinical diagnosis, as well as the value of FFPE archives for biomedical research. Proteins present in FFPE samples remain stable for long periods, making them particularly useful for retrospective analysis.^112^ Despite the advantages of FFPE samples, there are limitations associated with their use. One such limitation is the potential for mutual crosslinking of nucleic acids and formaldehyde. Formaldehyde can covalently crosslink nucleic acids and proteins, causing fragmentation of nucleic acids and making the extraction of high‐quality RNA and DNA challenging. In contrast, biopsies are typically taken directly from the tumor site, providing a more precise representation of the virus's presence in the tissue. Therefore, biopsies may be a more reliable sample type for detecting the virus.

This study aimed to investigate the prevalence of EBV associated with GC from 1980 to 2022, employing meta‐regression analysis. The rationale for conducting this analysis was due to the prevalence of global viral pandemics during this time, which may have had an adverse effect on the virus's behavior. The results of the analysis indicated that the prevalence of EBV associated with GC remained relatively constant over the four decades, suggesting that viral pandemics or other time‐related factors are not significant influencers of the virus's behavior.

The findings of this study suggest that there is no meaningful correlation between the development criteria of various countries and the prevalence of EBV associated with GC. However, further in‐depth investigations need to be conducted among countries with differing development indicators to validate the results of this study. Several studies^19^, ^22^, ^96^ The results indicate that ISH‐EBER is considered the most reliable method for detecting EBV due to its ability to identify EBER1 signals in the nucleus of nearly all cancer cells in EBV‐associated gastric carcinoma. Therefore, ISH‐EBER can be considered the gold standard method for detecting EBV.^113^ Certain researchers have solely relied on studies that have diagnosed the presence of EBV through the in situ hybridization technique to evaluate the overall prevalence of EBV associated with GC. They argue that the sensitivity and specificity of each diagnostic method vary, and the outcomes derived from combined data are not reliable. On the other hand, Zheng et al. believe this, stating that EBV testing using ISH is both time‐consuming and expensive, and there are no cost or time savings associated with it.^114^ After analyzing the time‐consuming and expensive nature of ISH‐EBER‐1, as reported by Zheng et al. we have chosen to analyze all studies that have employed various EBV detection methods, including PCR, immunohistochemistry, and different in situ hybridization techniques, even those that do not utilize in situ hybridization for coding RNAs of EBV in tissues. It is important to note that while PCR is a cost‐effective and straightforward method for detecting EBV infection, its low sensitivity makes it vulnerable to false‐positive results. Deyhimi et al.^115^ and Chen et al.^116^ reported that one explanation for the low sensitivity of PCR is that it can detect the presence of the EBV genome not only in tumor cells but also in nontumor cells like memory cells and lymphocytes. However, Chen et al. have argued that because more than 90% of people carry the virus, their lymphocytes may also contain the EBV genome, making it difficult for PCR to distinguish between cancer cells and infiltrating lymphocytes in the tumor stroma. Consequently, it is not possible to determine the origin of the amplified EBV genome. While PCR‐based methods are more sensitive than the gold standard ISH for detecting EBV, they are less specific.^115^, ^116^ It is important to acknowledge that certain methods may have limited research studies conducted on them. As a result, it is necessary to conduct further extensive research studies to obtain conclusive results regarding these methods.

The current study has several limitations that should be considered. Firstly, some important risk factors, such as the socioeconomic status and age of the patients, were not examined due to the unavailability of relevant information. Secondly, different diagnostic tests were used to diagnose EBV‐associated GC, which varied in sensitivity and specificity values. Thirdly, the study only included English‐language studies and a limited number of databases, which may have restricted access to articles and studies published in other languages or databases. Fourthly, we did not consider gray literature, potentially resulting in some missing data. Finally, the subjective nature of quality assessment, with a set inclusion threshold, introduces potential bias as different reviewers may evaluate the same study differently. Therefore, the researchers recommend that these limitations be taken into account, and further research should be conducted to investigate the relationship of EBV with other microorganisms like Helicobacter pylori, the association of EBV with cancer stages, potential risk factors, age, genetic and epigenetic factors. Despite numerous studies conducted worldwide to investigate the prevalence of EBV‐associated GC, there are still research gaps, and many regions of the world have not been evaluated, which may affect the estimated prevalence. Therefore, the study's results should be interpreted with caution.

## 5 CONCLUSION

The present study estimated an overall prevalence of 9.5% of EBV in GC samples, indicating that EBV infection is a significant factor in GC development. However, it is important to note that this does not necessarily imply that EBV infection directly causes GC. To obtain a more comprehensive understanding of the association between the virus and GC, it is advisable to conduct extensive epidemiological studies that explore various aspects of their relationship. The findings of our study hold the potential to facilitate the development of systematic screening programs aimed at controlling and ultimately preventing the impact of this parameter on GC occurrence. Given that males have twice the prevalence of EBV‐associated GC compared to females, it is recommended that other researchers investigate with more sensitivity the genetic, hormonal, and environmental factors that may be involved in this process to provide a clearer perspective on this area. Given the time‐consuming and costly nature of the ISH test and the absence of a generic alternative test that confirms EBV with the same accuracy as ISH, the development of a more cost‐effective and accessible tool for confirming EBV can be a crucial and effective step in reducing unnecessary costs and saving patients' time. Consequently, we suggest that researchers in the biomedical sciences devote more attention to this area.

## AUTHOR CONTRIBUTIONS

**Saman Dokanei**: Data curation. **Dariush Minai‐Tehrani**: Resources. **Mohsen Moghoofei**: Data curation. **Mosayeb Rostamian**: Formal analysis.

## CONFLICT OF INTEREST STATEMENT

The authors declare no conflict of interest.

## TRANSPARENCY STATEMENT

The lead author Mosayeb Rostamian affirms that this manuscript is an honest, accurate, and transparent account of the study being reported; that no important aspects of the study have been omitted; and that any discrepancies from the study as planned (and, if relevant, registered) have been explained.

## Data Availability

The authors confirm that the data supporting the findings of this study are available within the article [and/or] its supplementary materials.

## ENDNOTES

* PRISMA: Preferred Reporting Items for Systematic Reviews and Meta‐Analysis.

† CMA: Comprehensive Meta‐analysis.
